# TLQP-21 Protects Human Umbilical Vein Endothelial Cells against High-Glucose-Induced Apoptosis by Increasing G6PD Expression

**DOI:** 10.1371/journal.pone.0079760

**Published:** 2013-11-21

**Authors:** Wei Zhang, Chao Ni, Jie Sheng, Yanyin Hua, Jiangbo Ma, Lijun Wang, Yu Zhao, Yubo Xing

**Affiliations:** 1 Department of Endocrinology, Zhejiang People's Hospital, Hangzhou, Zhejiang, China; 2 Cancer Institute (Key Laboratory of Cancer Prevention and Intervention, National Ministry of Education, Provincial Key Laboratory of Molecular Biology in Medical Sciences), The Second Affiliated Hospital, Zhejiang University School of Medicine, Hangzhou, China; Bristol Heart Institute, University of Bristol, United Kingdom

## Abstract

Hyperglycemia causes oxidative stress that could damage vascular endothelial cells, leading to cardiovascular complications. The *Vgf* gene was identified as a nerve growth factor-responsive gene, and its protein product, VGF, is characterized by the presence of partially cleaved products. One of the VGF-derived peptides is TLQP-21, which is composed of 21 amino acids (residues 556–576). Past studies have reported that TLQP-21 could stimulate insulin secretion in pancreatic cells and protect these cells from apoptosis, which suggests that TLQP-21 has a potential function in diabetes therapy. Here, we explore the protective role of TLQP-21 against the high glucose-mediated injury of vascular endothelial cells. Using human umbilical vascular endothelial cells (HUVECs), we demonstrated that TLQP-21 (10 or 50 nM) dose-dependently prevented apoptosis under high-glucose (30 mmol/L) conditions (the normal glucose concentration is 5.6 mmol/L). TLQP-21 enhanced the expression of NAPDH, resulting in upregulation of glutathione (GSH) and a reduction in the levels of reactive oxygen species (ROS). TLQP-21 also upregulated the expression of glucose-6-phosphate dehydrogenase (G6PD), which is known as the main source of NADPH. Knockdown of G6PD almost completely blocked the increase of NADPH induced by TLQP-21, indicating that TLQP-21 functions mainly through G6PD to promote NADPH generation. In conclusion, TLQP-21 could increase G6PD expression, which in turn may increase the synthesis of NADPH and GSH, thereby partially restoring the redox status of vascular endothelial cells under high glucose injury. We propose that TLQP-21 is a promising drug for diabetes therapy.

## Introduction

The *Vgf* gene was first identified as a nerve growth factor-responsive gene in PC12 cells [1], which encodes a 617 (rat, mouse) or 615 (human) amino acid protein [2]. *VGF* is mainly expressed in pituitary and some peripheral endocrine cells, including gastroenteric endocrine cells, adrenal medulla cells and pancreatic β cells. VGF has been reported to play multiple roles in regulating energy homeostasis, metabolism and synaptic plasticity [3], [4]. Current knowledge about VGF is mainly derived from experiments with animals. For example, VGF-deficient mice displayed decreases in circulating insulin levels and reduced islet cell mass [5]. Recently, more specific studies of VGF have focused on its post-translational cleavage products. One of its best characterized peptides is TLQP-21 (amino acid residues 556 to 576) [6]. At present, several biological functions of this peptide have been identified, including negative effects on body weight via increased energy expenditure and control of gut functioning [6]–[8], a gastroprotective role against ethanol injury via increased levels of constitutive nitric oxide (NO) and prostaglandin E2 (PGE2) [9], and possible indirect regulation of pancreatic exocrine secretion [10].

However, most of these reports were based on mammalian neuroendocrine systems, excluding human beings. In fact, some studies have shown that TLQP-21 has direct anti-apoptotic effects on cells. Severini et al. (2008) reported that TLQP-21 could prevent cerebellar granule cell death induced by serum and potassium deprivation. Recently, Stephens et al. (2012) found that TLQP-21 plays an important role in diabetes. Interestingly, it shares many properties, such as potentiating glucose-stimulated insulin secretion, improving glycemic control, and reducing islet cell apoptosis, with GLP-1 receptor agonists, which have been widely used in diabetes therapy. In addition, TLQP-21 lacks some side effects, such as nausea, vomiting, abdominal distension, and poor appetite, of glucagon-like peptide-1 (GLP-1), which is a type of incretin hormone secreted by L-cells in the intestine that helps to stimulate insulin secretion, receptor agonists [11]. These side effects are common and may cause the termination of treatment [12] . These findings led us to investigate whether TLQP-21 could parallelize the effects of GLP-1 in diabetes therapy. Recent research has shown the direct protective effect of GLP-1 on vascular endothelial cells under high-glucose stimulation [13]–[15], but whether TLQP-21 has such direct effects on vascular endothelial cell in diabetes is still unknown. In the present study, we attempted to investigate whether TLQP-21 has direct anti-apoptotic effects on HUVECs with high glucose injury, and if so, to uncover the relevant mechanisms of these effects.

## Results

### TLQP-21 attenuates the cytotoxic effect of high glucose on HUVECs

**Figure 1:**
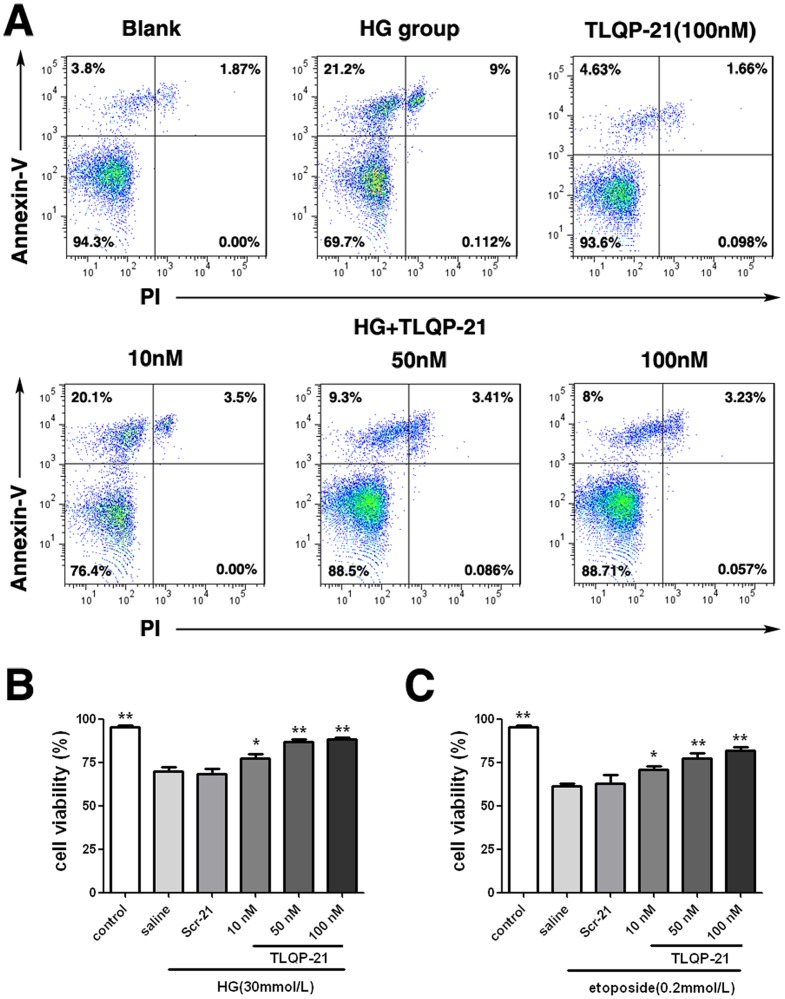
The protective effect of TLQP-21 to HUVECs with cytoxic injury. (A) Annexin-V-PI staining to assess the apoptotic ratios of HUVECs with TLQP-21 treated alone or under high glucose (HG) concentration (30 mmol/L) incubation for 72 h with or without TLQP-21(10–100 nM). (B) Cell viability of HUVECs under HG concentration (30 mmol/L) incubation for 72 h with or without TLQP-21(10–100 nM) protection.* p<.05,** p<.01 vs. saline treated group. (C) Cell viability of HUVECs with etoposide (0.2 mM) treatment for 72 h with or without TLQP-21(10–100 nM) protection. * p<.05, ** p<.01 vs. saline treated group. All data were collected from three independent experiments and are presented as the mean ±SD.

First, we assessed the cytotoxic effect of high glucose (30 mmol/L) on HUVECs by FCM. As expected, high-glucose incubation for 72 h clearly induced apoptosis of the HUVECs (Figure 1A). We then determined whether TLQP-21 could protect cells from this injury. For this purpose, HUVECs were treated with high glucose and co-incubated with 10, 50, or 100 nM TLQP-21. Then, the cells were dispersed, and the proportions of apoptotic cells were determined by Annexin-V/PI staining. The results showed that TLQP-21 markedly decreased the apoptotic proportion in a dose-dependent manner (Figure 1A, B). Because a recent research report found that the protective effect of TLQP-21 on pancreatic beta-cells was nonspecific [2], we also applied etoposide (0.2 mM), a DNA damage-inducing agent, to HUVECs, followed by TLQP-21 treatment. The results revealed that the cytotoxic effects of etoposide could also be significantly attenuated by TLQP-21 (Figure 1C).

### TLQP-21 increases NAPDH expression in HUVECs

**Figure 2:**
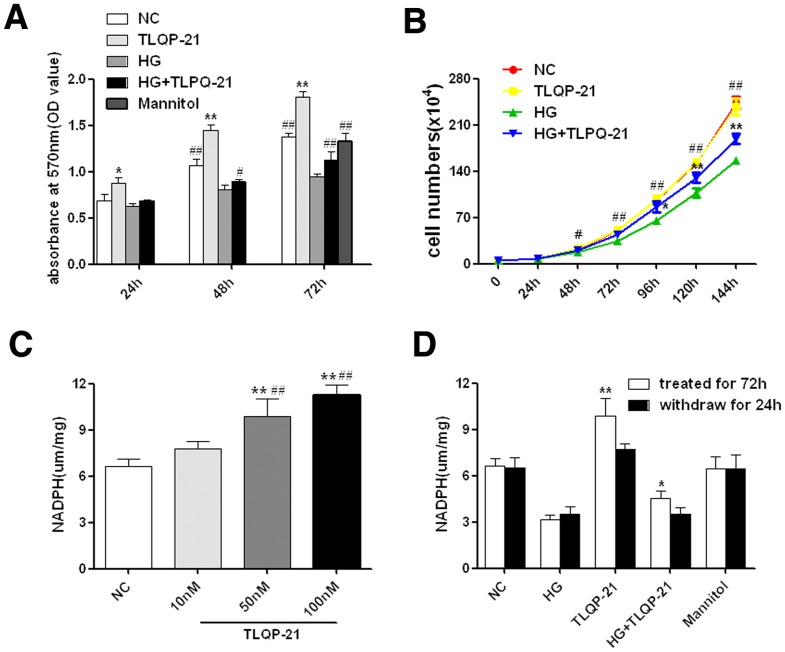
TLQP-21 increases the NADPH level without promoting cell growth. (A) The absorbance value of MTS assay with different treatments; the mannitol group was set as hyperosmosis control. *p<.05, **p<.01 vs. NC group; ^#^ p<.05, ^##^p<.01 vs. HG group. (B) Cell proliferation curve of HUVECs with different treatments. ^#^p<.05, ^##^p<.01 NC&TLQP-21 group vs. HG group; *p<.05, **p<.01 HG+TLQP-21 group vs. HG group. (C) NADPH level of HUVECs with different treatments. **p<.01 vs. NC group; ^##^p<.01 vs. TLQP-21(10 nM) group. (D) Comparison of the NADPH level of HUVECs with and without TLQP-21(50 nM) treatment; the mannitol group was set as a hyperosmosis control. *p<.05, *p<.01 vs. TLQP-21 withdraw group. All data were collected from three independent experiments and are presented as the mean ±SD.

We also performed MTS assays to detect the effect of TLQP-21 on cell proliferation. The absorbance values of cultures treated with TLQP-21 (50 nM) were significantly higher than that of cultures incubated either with normal or high glucose, and, as expected, were decreased in the high-glucose treatment group without TLQP-21 (Figure 2A). This finding motivated us to investigate whether TLQP-21 promotes cell growth. After quantitatively analyzing the cell numbers, the growth curves revealed that TLQP-21 had no effect on cell proliferation rates (Figure 2B). Because the absorbance value of the MTS assay mainly depends on the conversion of MTS to formazan, which relies on the NADPH/NADH level, we hypothesized that TLQP-21 might increase the NADPH/NADH activity. In a subsequent experiment, we found that the NADPH level in TLQP-21-treated cells was greatly increased in a dose-dependent manner compared to the control (Figure 2C), and high-glucose incubation alone led to a marked decrease in NADPH level (Figure 2D). However, after withdrawal of TLQP-21 for 24 h, the NADPH levels quickly returned to normal (Figure 2D).

### TLQP-21 upregulates GSH and decreases ROS production

**Figure 3:**
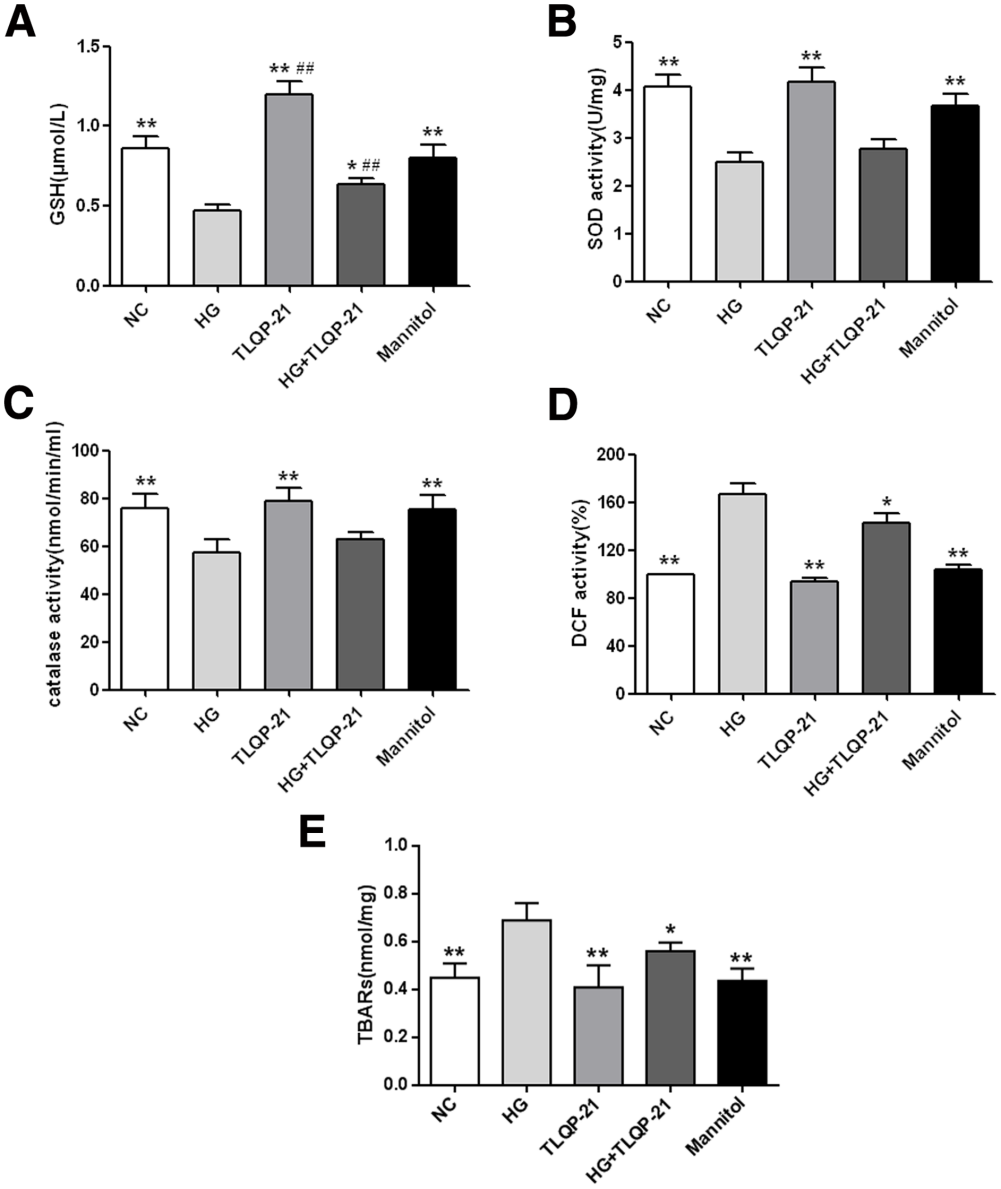
TLQP-21 treatment (72 h) protects the HUVECs from oxidative stress by intercellularly rebalancing the redox state. (A) GSH level of HUVECs with different treatments. (B) SOD activity of HUVECs with different treatments. (C) Catalase activity of HUVECs with different treatments. (D) DCF intensity presents the ROS levels of HUVECs with different treatments. (E) TBARs level of HUVECs with different treatments. *p<.05, **p<.01 vs. HG group; ^##^p<.01 vs. NC group. All data were collected from three independent experiments and are presented as the mean ±SD.

The glutathione system is one of the major antioxidant pathways and requires NADPH for GSSG to GSH conversion. Because TLQP-21 increased the NADPH level, we evaluated its effect on the GSH/GSSG system. The results indicated that high glucose markedly impaired the GSH level while TLQP-21 (50 nM, 72 h) treatment had a pronounced rescue function (Figure 3A). We then evaluated the effect of TLQP-21(50 nM, 72 h) on other important anti-oxidant systems, and the results revealed a non-significant upregulation of catalase and SOD expression levels (Figure 3B, C).

Because it is widely accepted that increased ROS levels are one of the principle causes of endothelial cell function impairment in diabetes and because NADPH occupies a core position in the antioxidant system, we next determined the effect of TLQP-21 on redox balance. FCM analysis revealed that ROS levels were increased after high-glucose incubation and that TLQP-21 (50 nM, 72 h) partially attenuated this injury (Figure 3D). To further confirm the anti-oxidant effect of TLQP-21, we measured TBARs and found that the increase in oxidized lipids observed after high-glucose incubation was also significantly decreased by TLQP-21 (Figure 3E).

### TLQP-21 increases NADPH expression via its effect on G6PD

**Figure 4:**
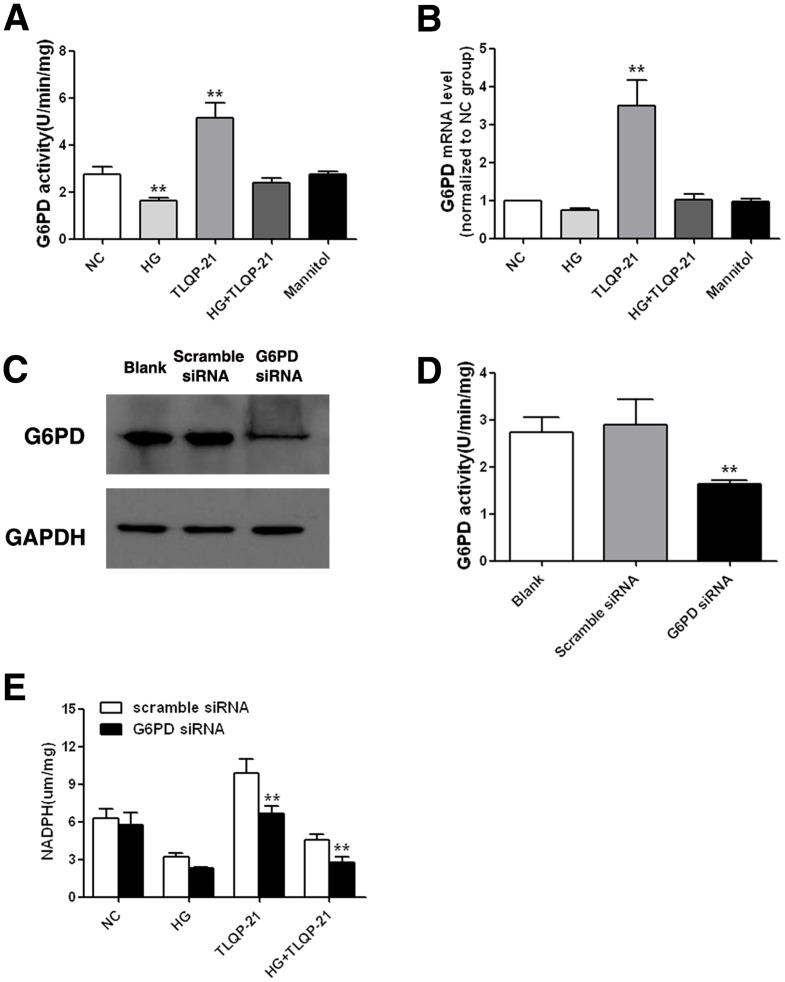
TLQP-21 increases NADPH expression, but depends on G6PD. (A) G6PD activity of HUVECs with different treatments. **p<.01 vs. NC group. (B) mRNA level of G6PD with different treatments. **p<.01 vs. NC group (C) Western-blot analysis shows that G6PD expression decreased with G6PD siRNA. (D) G6PD activity decreases with G6PD siRNA. **p<.01 vs. scramble siRNA group. (E) G6PD siRNA attenuates the upregulation of NADPH when treated with TLQP-21. **p<.01 vs. scramble siRNA group. All data were collected from three independent experiments and are presented as the mean ±SD.

Because TLQP-21 treatment did not increase the mRNA level of NADP+ (data not shown), which indicates that an indirect correlation exists, we hypothesized that TLQP-21 has an upstream effect on NADPH. Glucose 6-phosphate dehydrogenase (G6PD) is the first rate-limiting enzyme that participates in the pentose-phosphate pathway, and many studies have reported it to be the major source of NADPH. We found that both the transcriptional level and activity of G6PD was decreased upon high-glucose incubation and that they were increased when cells were treated with TLQP-21 (Figure 4A, B). To determine whether the upregulation of NADPH by TLQP-21 depends on its effect on G6PD, we downregulated G6PD expression with a small interfering RNA oligonucleotide. Then, we verified that G6PD expression and activity were significantly decreased by the siRNA (Figure 4C, D). The TLQP-21-mediated increase of NADPH expression was greatly weakened when transfected with G6PD siRNA (Figure 4E). These results suggest that the upregulation of NADPH by TLQP-21 was mainly dependent on its effect on G6PD.

## Discussion

VGF is expressed in many cell and tissue types, and proteolytic processing of VGF yields various peptides, including TLQP-21, TLQP-62, NERP-1 and -2 [3], [4], [16], [17]. Some studies have investigated the distribution of VGF-derived peptides in mammals, and the results revealed that VGFs were well represented outside of the central nervous system (CNS), in places such as the stomach and pancreatic islets [18], [19] . Interestingly, many experiments found stimuli from the external environment could affect specific subtypes of VGF-derived peptides. TLQP peptides have been shown to be decreased in fasting rats as compared with fed rats, demonstrating their close relationship with energy metabolism [19]. Similarly, previous studies have shown that TLQP-21 negatively affects energy balance and increases catabolism in adipose tissue [6], [8], [20]. Previous studies also demonstrated that intracerebroventricular infusion induced weight loss and protected against diet-induced obesity. More recently, TLQP-21 revealed direct anti-apoptotic and exocrine promotion effects on beta-cells. However, the mechanisms of these effects remain unclear. The earlier studies mentioned above suggest that TLQP-21 may have an anti-diabetes function; its complex effects suggest that this VGF-derived peptide has additional biological implications for diabetes therapy that remain to be discovered.

Cardiovascular dysfunction has been identified as the major direct cause of death in patients with diabetes mellitus, including both macrovascular and microvascular diseases [21]. High glucose increases ROS generation, mainly by mitochondria, and simultaneously decreases the level of intracellular antioxidants, resulting in dysfunction of vascular endothelial cells. For this reason, rebalancing the redox state could be an important goal for diabetes therapy. The major intracellular antioxidative systems are the glutathione system, the superoxide dismutases (SOD), catalase, and the thioredoxin (Trx) system [22], [23]. Our results provide the first evidence that TLQP-21 has a direct protective effect on human vascular endothelial cells under high-glucose-induced oxidative stress injury by upregulating GSH and decreasing ROS production. Interestingly, we found that TLQP-21 increases absorbance values in MTS assays. However, further experiments revealed that the cell proliferation rate was unchanged. Based on the principle mechanisms of MTS assays, we suspect that TLQP-21 increases NADPH levels.

NADPH is vital to the entire antioxidant system, mainly participating in electron transfer reactions to reduce oxidized elements and maintain the intracellular redox balance [24]. The majority of antioxidative systems are closely linked to NADPH. For example, the generation of glutathione reductase is dependent on NADPH [25]. NADPH may also bind to catalase to maintain its activity and to protect it from the toxic effects of hydrogen peroxide [26]. Moreover, although the production and function of SOD does not directly involve NADPH, its biological function requires either glutathione or catalase to convert hydrogen peroxide to a less toxic compound [27]. Together, these findings suggest that NADPH plays an indispensable role in the antioxidant system. Here, we show that TLQP-21 can increase NADPH levels, and, as hypothesized, can promote antioxidant effects, increasing the generation of GSH and decreasing ROS and TBAR production. These effects partially explain the mechanisms of the protective effect of TLQP-21 on vascular endothelial cells exposed to high-glucose injury. Neither catalase nor SOD were upregulated by TLQP-21 treatment, which, as indicated above, may imply that NADPH is not involved in their generation process.

Although TLQP-21 could increase the production of NADPH, the transcriptional level of NADP+ was not changed, which indicates that TLQP-21 could have an upstream effect on NADPH, but not a direct effect. Because it has been reported that high glucose decreases NADPH by impairing the activity of G6PD [28], [29], which is the primary source of NADPH, we investigated the effects of TLQP-21 on G6PD. Our results showed that G6PD expression could be upregulated by TLQP-21 and that knockdown of G6PD greatly attenuated the protective effect and upregulation of NADPH induced by TLQP-21. These results suggest that TLQP-21 may work indirectly through G6PD to affect the level of NADPH. *G6PD* is a housekeeping gene; its protein product works as the first and key enzyme in the pentose-phosphate pathway and is the sole source of NADPH generation in response to oxidative stress [30]–[32]. Abundant evidence suggests that upregulation of G6PD expression could dramatically improve the cellular redox status in diabetic or other high-glucose conditions. Overexpression of G6PD has been shown not only to affect GSH production but also to rescue the SOD and catalase activities without altering their protein expression [32], [33]; these results coincide with our present findings. Several studies have found that G6PD expression is regulated by cAMP and that inhibition of G6PD via activation of cAMP by high-glucose stimulation is dependent on protein kinase A (PKA) [34]. However, we did not find any change in PKA expression after TLQP-21 treatment (data not shown); therefore, further study is needed to elucidate the mechanism of its effect on G6PD.

Some reports suggested that G6PD has a “double-edged sword” effect, in that excess G6PD could produce abundant NADPH, fueling the activity of NADPH oxidases, which are known to play a dominant role in the production of superoxide, especially as a source of ROS [35], [36]. Gupte *et al*. [37] reported that high NADPH oxidase activity, generated from G6PD - derived NADPH, results in increased levels of superoxide in the failing hearts of patients with ischemic cardiomyopathy. Recently, Wang *et al*. [38] showed that over-expression of G6PD could result in the accumulation of pentose and pentosidine, which are associated with insulin resistance in obesity. The above evidence suggests that there are potential side effects of over-expression of G6PD and of its NADPH production. Here, we show that TLQP-21 could promote G6PD/NADPH generation and upregulate the intracellular anti-oxidant system without obvious side effects, suggesting its potential for diabetes therapy. However, some obstacles for clinical translation still remain, such as the fact that the effect of TLQP-21 did not appear to be long-lasting. Twenty-four hours after withdrawal, the NADPH level decreased rapidly (Figure 2D). Similar results *in vivo* have been reported by other researchers [2]. Such decreases may be caused by proteolytic cleavage events; these limitations indicate the need for future improvement in the formulation of TLQP-21 or a modification of the structure.

In conclusion, we found that vascular endothelial cells treated with TLQP-21 are protected against high-glucose stimulation, through increases in G6PD expression and NADPH, which enhance the glutathione system and decrease ROS levels. These effects suggest the importance of *in vivo* research of the use of TLQP-21 in diabetes therapy.

## Materials and Methods

### Cell cultures

Human Umbilical Vein Endothelial Cells were obtained from the Cell Bank of the Chinese Academy of Sciences (Shanghai, China). HUVECs were cultured in Endothelial Cell Medium (ECM, ScienCell) with 5% fetal bovine serum (FBS, Gibco, Invitrogen Corporation, Grand Island, NY, USA). The medium was changed every two days. Human-derived TLQP-21 (TLGPPSALAAAHTHHALPPSA) was obtained from Phoenix pharmaceuticals (USA), and the scrambled peptide Scr-21 (LRPSHTRPAHQSFARPLHRPA) was synthesized by Apeptide (Shanghai, China)

### Determination of cell viability and proliferation

Cell viability was tested with MTS dye according to the manufacturer's instructions (CellTiter96 Aqueous One Solution Assay, Promega, Madison, WI, USA). Cells were seeded into 96-well plates. After various treatments for 72 h, which were administered according to group sets, 20 µl of MTS reagent was added to each well, followed by incubation for 4 h. Absorbance was then measured at 490 nm, and the values were compared with those from control groups and reported as a relative percent of viabilities.

To determine proliferation rates, cells were plated at a density of 1.0×10^4^ per well in 24-well plates. After 12 h of seeding in a normal glucose concentration (5.5 mmol/L), cells of different groups were treated as indicated below, with each treatment performed in triplicate. Over the next 7 days, cells were harvested every 24 h, and living cells were quantified with a hemocytometer. Dead cells were identified by trypan blue staining.

### Apoptosis measurement by flow cytometry (FCM)

Apoptosis was assessed by Annexin-V/PI double staining (Annexin-V-FLOUS staining kit, Roche, Mannheim, Germany). Cells were harvested with trypsin and re-suspended in Annexin-V-FLOUS labeling solution for 15 min at room temperature. All samples were analyzed on a BD Aria II flow cytometer, and at least 10000 events per sample were collected.

### Measurement of NADPH, ROS, Thiobarbituric Reactive Substances (TBARS), and G6PD activity

Intracellular ROS levels were measured by the oxidation-sensitive fluorescent probe dye, 2′, 7′-dichlorodihydrofluorescein diacetate (H2DCFDA, Invitrogen Molecular Probes, *E*x/*E*m = 495 nm/529 nm). Cells were washed with PBS and then incubated with 20 µM H2DCFDA at 37°C for 30 min according to the manufacturer's instructions. Fluorescence intensities were detected with a flow cytometer (BD Aria II). NADPH measurement was performed with an EnzyChrom™ NADP/NADPH Assay Kit (Bioassay systems, Hayward, CA, USA). TBARs were assessed using the OxiSelect™ TBARs Assay Kit (Cell Biolabs, San Diego, CA, USA). G6PD activity was determined with a Glucose-6-Phosphate Dehydrogenase Activity Assay Kit (Biovision, Milpitas, CA, USA). All procedures were performed according to manufacturers' protocols.

### Measurement of Catalase, Glutathione/Glutathione disulfide (GSH/GSSG), and Superoxide dismutase (SOD) activity

The contents or activities of GSH/GSSG, catalase, and SOD in cells were determined by spectrophotometric methods according to the kit manufacturer's protocols (Biovision, Milpitas, CA, USA).

### siRNA transfection

Gene knockdown was achieved by transfecting HUVECs with G6PD small interfering RNAs (siRNAs) (negative control: 5′ - GCCCGCUUUGUAGCAUUCG - 3′, G6PD siRNA: 5′ - GCGUUAUCCUCACCUUCAATT - 3′, which were synthesized by Sangon Biotech, Shanghai, China) using Lipofectamine 2000 (Invitrogen), in accordance with the manufacturer's instructions. Knockdown efficiency was verified by quantitative reverse transcription polymerase chain reaction (qRT-PCR) and western blot 48 h after transfection.

### Quantitative reverse-transcription polymerase chain reaction (qRT- PCR)

Total RNA was extracted from cultured cells with Trizol (Invitrogen, CA, USA). A Taqman assay of G6PD was purchased from Applied Biosystems (Life Technologies Co., CA, USA) with the TaqMan Universal PCR Master Mix and analyzed with an ABI step-one-plus System (Applied Biosystems, Foster City, CA, USA).

### Western-blotting

Aliquots (20 µg) of total cell protein were resolved on 12% SDS­PAGE gels and transferred onto PVDF membranes. The membranes were incubated with the primary antibody of G6PD (Cell Signaling, Danvers, MA, USA) over night at 4°C, followed with HRP­conjugated secondary antibody incubation at room temperature for 1 h (Promega, Madison, WI, USA). The protein­antibody complexes were visualized using an enhanced Phototope TM­HRP Detection Kit (Cell Signaling) and exposed to a Kodak medical X-ray processor (Kodak, Rochester, NY, USA).

### Statistical analysis

All data were obtained from at least three independent experiments. The results are represented as the means ± SD. Statistical analyses were performed with GraphPad Prism 5, and all data were analyzed by one-way ANOVA tests and Student–Newman–Keuls (SNK) comparisons. *P* values less than 0.05 were considered statistically significant.
